# Measurement of stress-induced sympathetic nervous activity using multi-wavelength PPG

**DOI:** 10.1098/rsos.221382

**Published:** 2023-08-30

**Authors:** Radhagayathri Udhayakumar, Saifur Rahman, Dilpreet Buxi, Vaughan G. Macefield, Tye Dawood, Nicholas Mellor, Chandan Karmakar

**Affiliations:** ^1^ School of Information Technology Deakin University, Geelong 3225, Australia; ^2^ Baker Heart and Diabetes Institute, Melbourne, Australia; ^3^ Philia Labs Pty Ltd, Melbourne, Australia

**Keywords:** PPG, sympathetic arousal, Hand Grip exercise, Cold Pressor, AC amplitude, entropy

## Abstract

The onset of stress triggers sympathetic arousal (SA), which causes detectable changes to physiological parameters such as heart rate, blood pressure, dilation of the pupils and sweat release. The objective quantification of SA has tremendous potential to prevent and manage psychological disorders. Photoplethysmography (PPG), a non-invasive method to measure skin blood flow changes, has been used to estimate SA indirectly. However, the impact of various wavelengths of the PPG signal has not been investigated for estimating SA. In this study, we explore the feasibility of using various statistical and nonlinear features derived from peak-to-peak (AC) values of PPG signals of different wavelengths (green, blue, infrared and red) to estimate stress-induced changes in SA and compare their performances. The impact of two physical stressors: and Hand Grip are studied on 32 healthy individuals. Linear (Mean, s.d.) and nonlinear (Katz, Petrosian, Higuchi, SampEn, TotalSampEn) features are extracted from the PPG signal’s AC amplitudes to identify the onset, continuation and recovery phases of those stressors. The results show that the nonlinear features are the most promising in detecting stress-induced sympathetic activity. *TotalSampEn* feature was capable of detecting stress-induced changes in SA for all wavelengths, whereas other features (Petrosian, AvgSampEn) are significant (AUC ≥ 0.8) only for IR and Red wavelengths. The outcomes of this study can be used to make device design decisions as well as develop stress detection algorithms.

## 1. Introduction

‘Stress’ is an umbrella term representing experiences in which the environmental demands of a situation outweigh the individual’s perceived psychological and physiological ability to cope with it effectively [1]. Physical or psychological stressors activate the sympathetic nervous system, resulting in sympathetic arousal (SA). SA results in effector organ responses, which can be measured by physiological parameters such as blood pressure, skin blood flow and sweat release [2]. Chronic stress may cause physical, behavioural and/or neuropsychiatric disorders such as executive dysfunction, anxiety and depression; cardiovascular disorders, such as hypertension; metabolic disorders, such as obesity and type 2 diabetes mellitus; and sleep disorders, such as insomnia or excessive daytime sleepiness [3,4].

The World Health Organization (WHO) has declared stress as the ‘Health Epidemic of the Twenty-First Century’ [5]. Two in five Australians experience mental illness at least once in their lifetimes, while the recurrent expenditure on stress-related mental health services in Australia was estimated at $11 billion in 2019–2020 [6].

Despite widespread evidence that stress is important in managing health, defining and measuring ‘stress’ is complex, due to its being experienced at social, psychological and physiological levels [4]. Questionnaires are used on a routine basis to assess the exposure to stressors as well as the individual’s appraisal of these. While their credibility is supported by ample evidence and they are easy to administer, questionnaires do not capture the body’s response to stress in an objective and continuous manner, making it difficult to predict the onset of the aforementioned disorders that arise from chronic stress. Stress is associated with activation of the sympathetic nervous system, or SA [4,7,8], which has been the subject of much focus in creating objective and continuous markers using non-invasive wearable sensors [9,10].

These markers include analysis of heart rate (measured either from ECG or from finger blood flow) and its variability, skin conductance, pupil dilation and blood pressure, where the first and last markers have been a significant area of focus using photoplethysmography (PPG) at the finger, wrist and the ear [7]. To the best of our knowledge, only a limited number of laboratory studies have explored using the information in PPG signals as an indirect measure of SA [7,8], with the sensor in a wearable form factor e.g. wrist-strap.

Fei *et al.* explored the relation between PPG-based features (morphological and temporal) and emotional states [11]. Budidha & Kyriacou [12] have specifically studied the changes in pulse-transit time (PTT) and power spectral responses with varying phases of a Cold Pressor stress test. Although the study compared the performance of PTT measured from different body sites, they require both an electrocardiogram and a PPG signal to calculate PTT, increasing wearable device complexity for continuous monitoring of SA. In addition, although PPG signal can be acquired using LEDs (light emitting diodes) of multiple wavelengths (*λ*) such as Red (*λ* ∼ 690 nm), Infrared (IR) (*λ* ∼ 810 nm), Green (*λ* ∼ 530 nm) and Blue (*λ* ∼ 470 nm), the authors have explored the uses of IR PPG only. Even though Red and IR are the most commonly used wavelengths due to their deepest (around 2.5 mm) penetration capacity, the other lower wavelengths (blue and green) are also important to study since these are less prone to artefacts. Thus, each wavelength has its unique impact on PPG-based SA measurement [7,13,14] and should be analysed for making a knowledgeable design decision. Hence, further studies are necessary to establish the promise of using PPG-based methods to continuously monitor SA.

In this study, we extend the existing scope of PPG-based measurement of SA in three ways: (i) use a wide range of features from PPG-derived time series; (ii) use stressors other than a Cold Pressor test and (iii) analyse the performance for four wavelengths (Red, IR, Blue and Green). Existing studies mostly used low-frequency response of PPG signal, PPG morphology features, pulse rate and its variability and PTT features extracted from PPG signal to study SA. Pulse rate and its variability is an indicator of vagal tone, rather than SA. The suitability of PTT for single-point measurements is an investigation in itself.

To enable the development of a biomarker that is sensitive to SA alone, we investigate several statistical and nonlinear parameters of the pulsatile or AC component of PPG for studying SA. It is well established that the Cold Pressor test, which involves immersion of a hand in ice water, amplifies the direct constriction of cutaneous vessels, sympathetically mediated by an increase in cutaneous vasoconstrictor drive and is used widely for studying autonomic function [15,16]. In this study, we use isometric Hand-Grip exercise along with the Cold Pressor test to ensure the reliability of the responses found for PPG features during the Cold Pressor test. Finally, to the best of our knowledge, this study is the first to investigate the feasibility of using multi-wavelength PPG for estimating SA.

## 2. Methods

### 2.1. Data

#### 2.1.1. Acquisition

**Figure 1.**
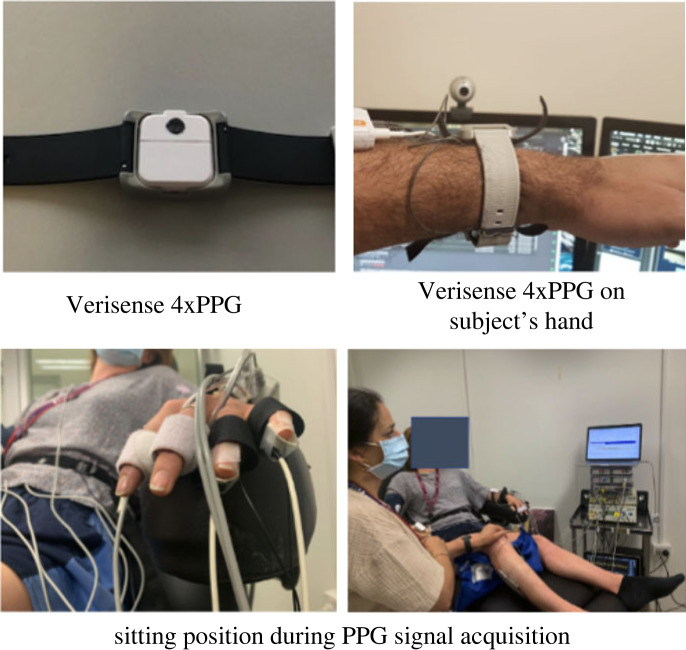
Experimental settings for non-invasive data collection of physiological parameters for right-handed subjects. Limb locations are switched for left-handed subjects.

Non-invasive physiological data (multi-wavelength PPG) were collected from 32 subjects (European and Asian ethnic backgrounds); 16 men and 16 women, all between 19 and 38 years of age, the mean age being 24. From each subject, multi-wavelength PPG (Red, Infrared, Green and Blue wavelengths) were obtained using Verisense Pulse^1^ device worn on the dorsal side (the upper side of the wrist) of the non-dominant arm. The data collection set-up (for the non-invasive signals) is shown in figure 1. Data from Verisense Pulse+ was collected at 100 Hz sampling rate and stored in Verisense’s Cloud server.

#### 2.1.2. Protocol

All data recordings commenced with the subject being seated on a reclined chair in a relaxed state (as seen in figure 1). PPG was first recorded during baseline condition (relaxed state) for 10 min before applying any stressors. Gradually, two instances of stressors (Hand Grip and Cold Pressor) were delivered in random order. Each stressor lasted for 2 min with a 5 min recovery period in between. The Hand Grip was applied at 30–35% maximal voluntary contraction, determined as the best of three maximal isometric contractions performed at the beginning of the experiment.

Following this, sensors were set up on the subject as shown in figure 1, and recordings were then obtained as follows:

(i) Baseline measures (relaxed state) were recorded for 10 min.
(ii) Measures were recorded as two stressors were delivered in random order
   (a) The Hand Grip test (2 min) was done using ADInstruments Grip Force Transducer. This allowed visualization of the participant’s maximal Hand Grip strength on a computer and showed the 30–35% strength range for the participant when they were undergoing the task so that they could maintain that threshold.
   (b) The Cold Pressor test (2 min) was done using a bucket filled with ice and water added to it. While we did not measure the temperature ourselves, the consensus is that ice water is about $4^{\circ }$ in temperature.
   (c) A 5-min recovery period between each stressor was also recorded.

Prior to data acquisition, complete ethics approval for the study and protocols was obtained from the Human Resources Ethics Committee of Western Sydney University (Ethics Reference: H11462). Participants gave informed consent upon participation.

### 2.2. Data preprocessing and analysis

Collected data were visually examined before analysis to confirm the quality of the signal, continuity of the signal over the data collection period and synchronization with event timings.

#### 2.2.1. Pulsatile and non-pulsatile components of PPG

The amplitude of the PPG signal at the peak corresponds to the blood volume during ventricular systole. The onset point of the PPG indicates the start of ventricular repolarization or diastole. This implies the amplitude of PPG at its onset gives us the pulsatile increase in cutaneous skin blood volume following ventricular diastole. This amplitude is also called the DC amplitude (direct current) or non-pulsatile component of the PPG. The pulsatile component of PPG is measured as the difference in blood volume between systole and diastole, which is in turn the difference in amplitudes of the peak and onset points, also called the AC amplitude (alternating current), obtained by high-pass filtering the PPG signal [14,17,18].

A peak-detection algorithm [19] was used to appropriately filter the PPG signal and then detect fiducial points from the same. The algorithm involves three major functions; (i) basic filtering of PPG and baseline detection, (ii) PPG peak detection and (iii) PPG onset detection.

**Figure 2.**
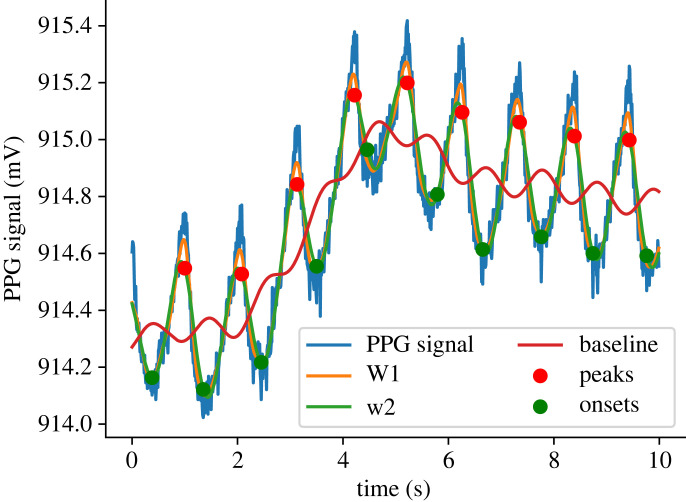
Example of PPG fiducial points detection.

*Filtering and baseline detection*. The initial step here is to find the inter-beat-interval (IBI) or frequency of heartbeats in the given PPG signal (blue solid line in figure 2), using power spectral analysis. Computing the power spectrum of the raw signal (PPG signal), IBI was estimated as the frequency corresponding to maximum power in the 0.8–3 Hz range of the spectrum. This frequency range translates to an average heart rate range of 50−180 beats min^−1^. The IBI is used as a control parameter in designing the filters (table 1).

**Table 1.** Variations (Mean ± s.d.) of features values extracted from pulsatile (AC) part of PPG signal in response to Cold Pressor event.

	(Mean ± s.d.)							
	Green				Blue			
AC feature	baseline	1st min	2nd min	recovery	baseline	1st min	2nd min	recovery
Mean	1.31 ± 0.88	1.13 ± 0.75	1.45 ± 1.06	1.42 ± 0.97	0.95 ± 0.67	0.79 ± 0.58	1.13 ± 1.10	1.01 ± 0.76
s.d.	0.90 ± 1.34	1.16 ± 1.35	1.12 ± 2.55	0.73 ± 0.61	0.67 ± 0.95	0.60 ± 0.48	1.34 ± 4.74	0.54 ± 0.43
Katz	2.43 ± 0.77	2.04 ± 0.72	2.64 ± 0.75	2.49 ± 0.53	2.40 ± 0.77	2.06 ± 0.63	2.44 ± 0.75	2.48 ± 0.56
Petrosian	1.04 ± 0.01	1.05 ± 0.02	1.04 ± 0.01	1.04 ± 0.01	1.04 ± 0.01	1.05 ± 0.02	1.01 ± 0.18	1.04 ± 0.01
Higuchi	1.90 ± 0.11	1.57 ± 0.78	1.90 ± 0.39	1.95 ± 0.15	1.91 ± 0.11	1.61 ± 0.82	1.89 ± 0.37	1.95 ± 0.15
SampEn	1.38 ± 0.50	—	—	1.48 ± 0.42	1.35 ± 0.51	—	1.59 ± 0.65	1.47 ± 0.49
TotalSampEn	496.57 ± 270.85	205.50 ± 141.66	275.10 ± 129.31	440.35 ± 215.43	372.06 ± 213.62	165.82 ± 125.25	227.82 ± 117.21	337.07 ± 182.12
AvgSampEn	0.30 ± 0.12	0.38 ± 0.09	0.40 ± 0.08	0.35 ± 0.07	0.28 ± 0.10	0.35 ± 0.13	0.39 ± 0.10	0.33 ± 0.07
	IR				Red			
	baseline	1st min	2nd min	recovery	baseline	1st min	2nd min	recovery
Mean	0.68 ± 0.34	0.59 ± 0.34	0.73 ± 0.40	0.69 ± 0.32	0.68 ± 0.34	0.59 ± 0.34	0.73 ± 0.40	0.69 ± 0.32
s.d.	1.34 ± 4.71	0.42 ± 0.36	0.56 ± 0.81	0.51 ± 0.50	3.61 ± 3.29	2.37 ± 2.34	1.76 ± 2.58	2.29 ± 1.91
Katz	2.14 ± 0.50	2.38 ± 0.72	2.50 ± 1.02	2.48 ± 0.75	1.67 ± 0.33	2.01 ± 0.92	2.14 ± 0.82	1.88 ± 0.71
Petrosian	1.04 ± 0.01	1.06 ± 0.02	1.05 ± 0.01	1.05 ± 0.01	1.04 ± 0.01	1.06 ± 0.01	1.06 ± 0.01	1.05 ± 0.01
Higuchi	1.98 ± 0.09	1.97 ± 0.55	2.06 ± 0.16	1.99 ± 0.08	2.07 ± 0.19	2.14 ± 0.48	2.16 ± 0.36	2.09 ± 0.26
SampEn	1.32 ± 0.52	—	—	1.50 ± 0.54	0.31 ± 0.47	0.89 ± 0.78	—	0.75 ± 0.79
TotalSampEn	302.19 ± 138.66	147.07 ± 74.32	180.52 ± 76.74	261.78 ± 109.15	256.28 ± 134.34	120.87 ± 47.91	124.63 ± 51.16	202.28 ± 86.76
AvgSampEn	0.28 ± 0.12	0.41 ± 0.09	0.44 ± 0.08	0.35 ± 0.07	0.26 ± 0.13	0.43 ± 0.09	0.42 ± 0.10	0.32 ± 0.07

Filtering has been done in three different stages as follows:

— Step 1: A centre median and centre moving-average filtering to smooth the signal. The smoothed signal (W1) is shown using a orange solid line in figure 2. This filtering was performed using a window size 0.2 * IBI.
— Step 2: A third order low pass Butterworth filter was used to remove any ectopic peaks and the output signal (W2) is shown as a green solid line in figure 2. The Butterworth filter used in this step was designed with a cut-off frequency of 1.5 * IBI.
— Step 3: In this step, a centre moving average was used for extracting the baseline and the output (baseline) is shown as a solid red line in figure 2. The window size used for moving-average filtering is 1.5 * IBI.

**Figure 3.**
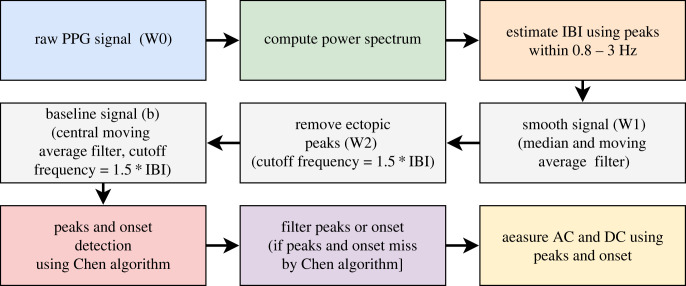
Overall data preprocessing steps from IBI to AC and DC value extractions.

**Figure 4.**
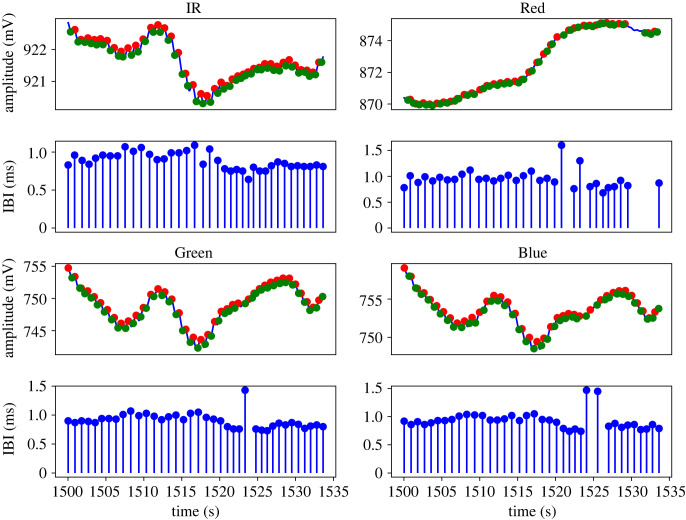
PPG onset and peaks for four wavelengths and their corresponding IBI in seconds. For the sake of plot visibility, the PPG signals are presented for specific events.

*PPG peak and onset detection.* An initial set of peaks is detected in the PPG signal (solid blue line) by locating the local maxima in every IBI. After detecting initial peaks, we used the same set of criteria on the heights of peaks and the inter-peak distances that were reported by Chen *et al.* [19] for identifying and removing false peaks. This step helps in retaining the true peaks and relocating them if necessary. Onsets, the local minima between two peaks on the PPG signal, were then detected using the true peaks (solid red circles in figure 2). Once the true peaks and onsets have been detected from a given PPG signal, the AC amplitude is obtained as the magnitudes of |peak-onset| and further filtered for outliers. The overall data preprocessing steps are shown in figures 3 and 4.

#### 2.2.2. Event signal generation and feature extraction

**Figure 5.**
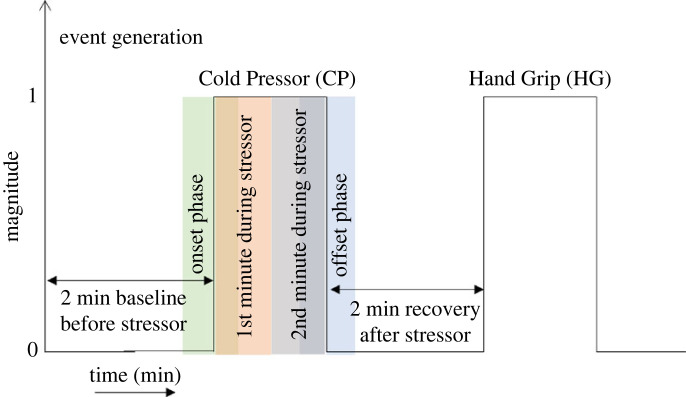
Event signal generated corresponding to the start and end of a stressor event. Features are extracted corresponding to four different durations related to the stressor; the first 2 min baseline period before the start of the stressor, the second 1st min during the stressor, the third 2nd min during the stressor and the final 2 min recovery period after the end of the stressor.

Corresponding to the onset, duration, and end of a stressor event (e.g. Cold Pressor, Hand Grip), a 1–0 (amplitude) event signal is generated and mapped to the length of the PPG signals acquired. The event signal has an amplitude of 1 during the period of a stressor and is 0 otherwise (figure 5).

Statistical, fractal dimensions and entropy-profile based features are extracted from the pulsatile AC component of the PPG signals. These features are extracted from four different time windows; each corresponding to a stressor phase as listed below:

— Baseline period, which is 2 min preceding onset of stressor
— First minute of stressor
— Second minute of stressor
— The recovery periods, which is 2 min immediately following end of stressor

The list of features extracted is given in table 2.

**Table 2.** Variations (Mean ± s.d.) of features values extracted from pulsatile (AC) part of PPG signal in response to Hand Grip event.

	(Mean ± s.d.)							
	Green				Blue			
AC feature	baseline	1st min	2nd min	recovery	baseline	1st min	2nd min	recovery
Mean	1.46 ± 1.21	1.29 ± 1.04	1.71 ± 1.45	1.53 ± 1.23	1.04 ± 0.90	0.93 ± 0.81	1.22 ± 0.10	1.09 ± 0.92
s.d.	0.62 ± 0.34	0.56 ± 0.42	0.84 ± 0.95	0.68 ± 0.54	0.44 ± 0.25	0.39 ± 0.26	0.56 ± 0.60	0.48 ± 0.37
Katz	2.58 ± 0.68	2.62 ± 0.95	2.10 ± 2.16	2.52 ± 0.64	2.57 ± 0.64	2.43 ± 0.58	−14.7 ± 96.82	2.54 ± 0.67
Petrosian	1.04 ± 0.01	1.04 ± 0.01	1.04 ± 0.02	1.04 ± 0.01	1.04 ± 0.01	1.04 ± 0.01	1.01 ± 0.19	1.04 ± 0.01
Higuchi	1.89 ± 0.10	1.93 ± 0.16	1.87 ± 0.40	1.91 ± 0.12	1.89 ± 0.09	1.94 ± 0.18	1.82 ± 0.52	1.91 ± 0.11
SampEn	1.49 ± 0.46	1.66 ± 0.58	1.60 ± 0.70	1.51 ± 0.33	1.49 ± 0.42	1.64 ± 0.60	—	1.48 ± 0.39
TotalSampEn	460.16 ± 241.82	251.25 ± 108.19	285.54 ± 160.11	441.12 ± 232.38	347.32 ± 197.49	192.84 ± 102.36	229.32 ± 152.31	329.75 ± 195.37
AvgSampEn	0.30 ± 0.09	0.38 ± 0.09	0.39 ± 0.09	0.35 ± 0.07	0.30 ± 0.09	0.37 ± 0.08	0.38 ± 0.12	0.33 ± 0.07
	IR				Red			
	baseline	1st min	2nd min	recovery	baseline	1st min	2nd min	recovery
Mean	0.66 ± 0.39	0.54 ± 0.30	0.69 ± 0.35	0.69 ± 0.39	0.71 ± 0.41	0.75 ± 0.73	0.69 ± 0.49	0.65 ± 0.35
s.d.	0.34 ± 0.21	0.25 ± 0.14	0.48 ± 0.39	0.36 ± 0.20	2.45 ± 1.75	2.22 ± 2.53	1.91 ± 2.15	1.82 ± 1.88
Katz	2.66 ± 0.69	2.94 ± 0.97	2.19 ± 0.64	2.61 ± 0.73	1.80 ± 0.55	2.36 ± 1.34	1.88 ± 0.68	1.95 ± 0.65
Petrosian	1.04 ± 0.01	1.05 ± 0.01	1.05 ± 0.01	1.05 ± 0.01	1.05 ± 0.01	1.06 ± 0.01	1.06 ± 0.02	1.05 ± 0.01
Higuchi	1.97 ± 0.08	2.05 ± 0.18	1.82 ± 0.73	1.98 ± 0.09	2.02 ± 0.19	2.13 ± 0.49	2.08 ± 0.61	2.05 ± 0.22
SampEn	1.61 ± 0.42	—	—	1.71 ± 0.42	0.57 ± 0.82	0.93 ± 0.89	—	0.94 ± 0.86
TotalSampEn	277.62 ± 139.84	152.38 ± 67.22	161.88 ± 94.30	266.24 ± 114.39	241.12 ± 158.05	106.33 ± 39.51	123.87 ± 55.53	203.52 ± 89.35
AvgSampEn	0.32 ± 0.09	0.40 ± 0.08	0.41 ± 0.09	0.35 ± 0.06	0.28 ± 0.10	0.39 ± 0.10	0.41 ± 0.13	0.33 ± 0.07

#### 2.2.3. Statistical features

Linear time domain measures, namely Mean and s.d., are computed on the signal. While the Mean summarizes the data for interpretation, s.d. show us the spread of data around the Mean. Given the AC time-series signal *X*(*n*) = {*x*_1_, *x*_2_, …, *x*_*N*_} of length *N*, then Mean and s.d. can be computed using equations (2.1) and (2.2):

2.1 $$\mathrm{Mean}=\frac{1}{N}\begin{array}{c} N\\ \sum \\ i=1 \end{array}x_{i}$$

and

2.2 $$s.d.=\sqrt{\frac{1}{N}\begin{array}{c} N\\ \sum \\ i=1 \end{array}{\left(x_{i}-\mathrm{Mean}\right)}^{2}}.$$

#### 2.2.4. Fractal dimensions

Fractal dimensions (FDs) are ratios that quantify the complexity contained in a signal by measuring the changes in signal pattern with the scale (dimension) of observation.

Among available fractal dimension analysis techniques, we have selected three popularly used methods (Petrosian, Katz and Higuchi) for physiological signal analysis [20,21]. Esteller *et al.* [22] have compared Katz, Higuchi and Petrosian for different window sizes, and observed that 150 (less than sample frequency (250 Hz)) to 8000 samples. They have concluded that careful selection of the FD algorithm is necessary for individual applications with respect to data length, noise level and FD range. Since we have a smaller data length (approx. 120 samples/window), we have just directly applied the method to see their performance in differentiating sympathetic activation after applying stressors.

The first, in this study Katz, can be defined as follows:

2.3 $$\mathrm{Katz}=\frac{\log(S/M)}{\log(d/M)}$$

where, *S* is the sum and *M* is the mean of the Euclidean distances between successive samples of the signal and *d* is the maximum distance between the first sample and any other sample of *x*(*n*) [23].

The Petrosian algorithm [22,24] computes fractal dimension from the binary equivalent of a given signal *X*(*n*). The binary sequence from the signal can be generated in multiple ways [22]. For instance, taking a sample window of the signal and computing the window mean, say *Avg*, the binary sequence will take the value ‘1’ (corresponding to a sample of *X*(*n*)) if the individual sample value is greater than *Avg* and take ‘0’ if not. From such a binary equivalent, the Petrosian dimension is calculated as

2.4 $$\mathrm{Petrosian}=\frac{\log_{10}n}{\log_{10}n+\log_{10}(n/\left(n+0.4N_{\Delta }\right))}$$

where, *n* is the length of the sequence (same as the signal) and *N*_Δ_ is the number of sign changes (ones to zeros and vice-versa) in the generated binary sequence.

From the signal {*x*(*n*) : 1 ≤ *n* ≤ *N*}, let vectors given by

$$X_{k}^{m}=\left\{x(m+ik)\;:\;0\leq i\leq \frac{N-m}{k}\right\}$$

where $k\;\epsilon \;[1,k_{\max}]$ and m ϵ [1,k]. *k*_max_ is a parameter that is greater than or equal to 2. The length of each $X_{k}^{m}$ is then calculated as

$$L_{m}(k)=\frac{N-1}{[\left(N-m\right)/k]k^{2}}\sum_{i=1}^{\left(N-m\right)/k}|x(m+ik)-x(m+(i-1)k)|.$$

From this, an average estimate of *L*_*m*_(*k*) across *m*, for *k* sets, is given by

$$\left\langle L(k)\right\rangle =\frac{1}{k}\sum_{m=1}^{k}L_{m}(k).$$

The fractal dimension Higuchi is then given by [23,25]

2.5 $$\mathrm{Higuchi}=\frac{\log\left\langle L(k)\right\rangle }{-\log k}.$$

#### 2.2.5. Entropy features

Entropy estimates quantify signal complexity by looking if there are similar patterns in the signal at multiple scales of observation. The level of self-similarity is what entropy measures try to capture. The physiological signals being considered are highly complex and nonlinear in nature. They might exhibit different levels of self-similarity at different scales. This is the reason entropy is being used in this work. The entropy methods (entropy profiling) that we have used in this study are applicable to much smaller data length starting from 50 samples. The reliability of this measure for such small samples has been shown in our previous publications [26–28].

*Sample entropy (SampEn).* SampEn [29] is a self-match that is avoided in the estimation, unlike ApEn. A time series {*x*(*n*) : 1 ≤ *n* ≤ *N*} is divided into (*N* − *m*) overlapping vectors, each of length *m*, given by

$$\{X_{i}^{m}\;:\;1\leq i\leq (N-m)\},\;\mathrm{where}$$

$$X_{i}^{m}=\{x(i+k)\;:\;0\leq k\leq m-1\}$$

$C_{i}^{m}(r)$ is then the probability of a vector $X_{\;j}^{m}$ to lie within a distance *r* of the vector $X_{i}^{m}$, 1 ≤ *j* ≤ (*N* − *m*), *j* ≠ *i*, distance given by

$$d_{ij}^{m}=\{\max|X_{i}^{m}-X_{\;j}^{m}|\;:\;1\leq j\leq (N-m),j\neq i\}.$$

Then,

2.6 $$\mathrm{SampEn}(N,m,r)=\ln\frac{\Phi^{m}(r)}{\Phi^{m+1}(r)}$$

where $\Phi^{m}(r)=(1/\left(N-m\right))[i=1]N-m\sum C_{i}^{m}(r)$.

For all experiments conducted in this study, ApEn and SampEn are evaluated at an *r* value of 0.15 * s.d. of signal and an *m* value of 2.

Instead of choosing a single value of tolerance *r* to estimate SampEn, we could use a complete set of data driven *r* values (*r* : 1 ≤ *r* ≤ *q*) and generate a profile of SampEn values (SampEn(*r*)). This method of entropy profiling was introduced in 2018 by Udhayakumar *et al.* [27,30].

2.7 $$\mathrm{SampEn}(r)=\ln\frac{\Phi^{m}(r)}{\Phi^{m+1}(r)};\;1\leq r\leq q.$$

*Total Sample Entropy (TotalSampEn).* TotalSampEn is calculated by adding up all the individual values of SampEn(*r*) along the SampEn profile of a signal;

2.8 $$\mathrm{TotalSampEn}=\sum_{r=1}^{q}\mathrm{SampEn}(r).$$

*Average Sample Entropy (AvgSampEn)*. AvgSampEn is calculated by finding the mean of all the individual values of SampEn(*r*) along the SampEn profile of a signal;

2.9 $$\mathrm{AvgSampEn}=\frac{1}{q}\sum_{r=1}^{q}\mathrm{SampEn}(r).$$

### 2.3. Statistical analysis

In our study, we have used two statistical test parameters; the *p*-value and area under the ROC (receiver operating characteristic) curve in order to test the efficiency of regularity measures in signal classification. The *p*-value was calculated using non-parametric Mann–Whitney *U* test, since we have smaller number of samples we have selected the non-parametric approach over alternative methods. *p* can take values from 0 to 1 and in this study, we considered *p* < 0.05 statistically significant, rejecting the null hypothesis, i.e. the samples are not from the same distributions or the values are significantly different between two groups.

The area under the ROC curve (AUC) is the probability that a classifier ranks a randomly chosen instance *X* higher than a randomly chosen instance *Y*, *X* and *Y* being samples taken from two independent populations. An AUC value of 0.5 indicates that the distributions of the features are similar in the two groups with no discriminatory power. Conversely, an ROC area value of 1.0 would mean that the distribution of the features of the two groups do not overlap at all. Matlab R2019b Statistics toolbox was used to perform all statistical operations.

## 3. Results

The Mean ± s.d. amplitude of the extracted features corresponding to the phases of Cold Pressor and Hand Grip events for all four wavelengths of PPG (Green, Blue, IR and Red) is tabulated in tables 1 and 2, respectively.

### 3.1. Statistical features

**Figure 6.**
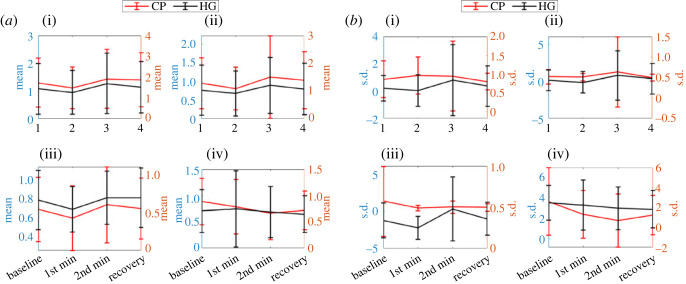
Pattern of (*a*) Mean and (*b*) s.d. feature changes (during 1st min, 2nd min, recovery phase) for HG and CD stressor across the Green (i), Blue (ii), IR (iii) and Red (iv) wavelength.

The average amplitude of Mean AC feature decreased during the 1st min of both stressors applied but increased during the 2nd min and decreased again during recovery for the green wavelength. However, these variations between different phases (baseline versus 1st min, 1st min versus 2nd min and 2nd min versus recovery) are not statistically significant (table 3 and figure 6*a*). A similar pattern was found for the Mean feature for the blue wavelength and the changes between different phases remains statistically insignificant (table 3). By contrast to Green and Blue wavelength, the Mean feature showed different patterns of changes for IR and Red wavelengths for two different stressors (CP and HG). Although the IR varies similarly to the Green and Blue wavelengths for CP, the amplitude decreased for the 1st min and increased for the 2nd min during HG stressor application and did not further decrease during the recovery phase. Interestingly, the differences in Mean feature between 1st min and 2nd min of HG stressor were statistically significant (table 3 and figure 6*a*), whereas all other changes remained statistically insignificant. Mean feature extracted from Red wavelength for CP stressor application showed a similar pattern as was found for Blue and Green wavelength, but it increased during 1st min of HG stressor application. Similar to Green and Blue wavelengths none of these differences in Mean values between phases are statistically significant (table 3 and figure 6*a*). Standard deviation (s.d.) of AC extracted from Blue and IR wavelengths showed similar patterns (decrease during 1st min, increase during 2nd min and decrease during recovery). However, for Blue wavelength such changes were statistically insignificant but for IR wavelength changes between 1st min and 2nd min of HG stressor was found significant (table 3 and figure 6*b*). By contrast, for Green wavelength, average s.d. value increased during 1st min of CP stressor then consistently decreased in subsequent phases. Whereas, for HG it decreased during 1st min, increased at 2nd min and then decreased again during the recovery phase. For the Red wavelength, for HG it showed a continuously decreasing pattern whereas, for CP stressor, it decreased till 2nd min of application of stressor and increased during recovery. However, differences in s.d. values between phases for Green and Red wavelengths are all statistically insignificant (table 3 and figure 6*b*).

**Table 3.** Statistical significance of PPG features to detect difference in phases of a Cold Pressor and Hand Grip test. Let us define the phase differences as follows; *A* = Baseline versus 1st min, *B* = 1st min versus 2nd min and *C*—2nd min versus Recovery. Let us define the magnitude of significance with the following symbols; $—*p* < 0.05, *—*p* < 0.01. The lower the value of *p*, the higher the statistical significance of separation between the phases.

	CP				HG			
AC feature/event	Green	Blue	IR	Red	Green	Blue	IR	Red
Mean	*ABC*	*ABC*	*ABC*	*ABC*	*ABC*	*ABC*	*AB*^$^*C*	*ABC*
s.d.	*ABC*	*ABC*	*ABC*	*ABC*	*ABC*	*ABC*	AB*C	*ABC*
Katz	*A*^$^B*C	*AB*^$^*C*	*ABC*	*ABC*	*ABC*	*ABC*	AB*C^$^	*ABC*
Petrosian	A*BC	*A*^$^*BC*	A*BC*	A*BC*	*A*^$^*BC*	*A*^$^*BC*^$^	A*BC^$^	A*BC
Higuchi	*ABC*	*ABC*	*A*^$^*BC*^$^	A*BC	*ABC*	*ABC*	*A*^$^*BC*	A*BC*
SampEn	*AB*^$^*C*	*AB*^$^*C*	*ABC*	A*BC	*ABC*	*ABC*	*ABC*	*ABC*
TotalSampEn	A*BC*	A*BC^$^	A*BC*	A*BC*	A*BC^$^	A*BC^$^	A*BC*	A*BC*
AvgSampEn	A*BC*	*A*^$^BC*	A*BC*	A*BC*	A*BC^$^	A*BC^$^	A*BC*	A*BC*

### 3.2. Fractal dimension features

**Figure 7.**
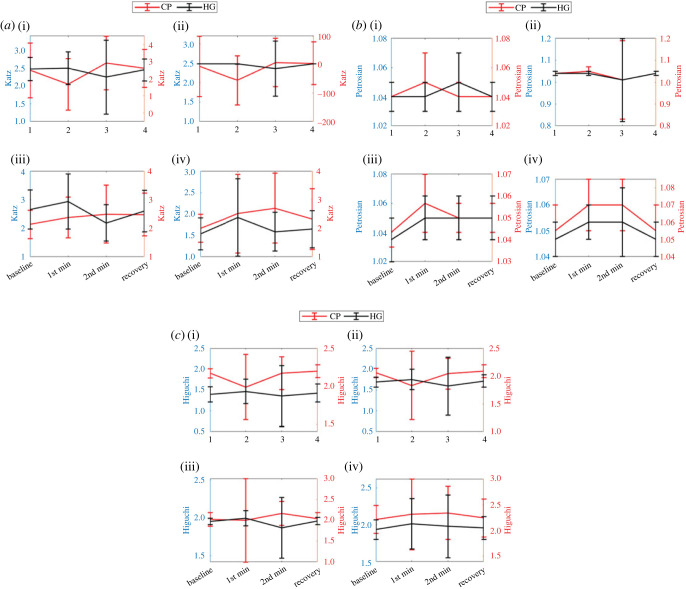
Pattern of (*a*) Katz, (*b*) Petrosian and (*c*) Higuchi feature changes (during 1st min, 2nd min, recovery phase) for HG and CD stressor across the Green (i), Blue (i), IR (iii) and Red (iv) wavelength.

The average Katz value showed a consistent pattern (increase, decrease and increase during 1st min, 2nd min and recovery phase, respectively) for HG stressor for all wavelengths. However, the complete opposite pattern (decrease, increase, decrease) was found for CP stressor for Green and Blue wavelengths. Average Katz feature extracted from IR and Red wavelength during CP stressor showed increase, increase and decrease pattern during 1st min, 2nd min and recovery phases, respectively. Changes in Katz feature values between 1st min and 2nd min of stressor were found statistically significant for phases for Green and Blue wavelength during CP stressor and IR wavelength for HG stressor (table 3 and figure 7*a*). The Green wavelength also showed statistically significant differences in Katz values between baseline and 1st min during CP stressor.

Average Petrosian value showed a consistent pattern (increase, decrease and increase during 1st min, 2nd min and recovery phase, respectively) for CP stressor for all wavelengths except Red. However, it has shown completely random patterns for HG stressor across all wavelengths. Interestingly, both stressors showed same pattern increase at 1st min and decrease at recovery phases for Red wavelength. Changes in Petrosian values were found statistically significant between baseline and 1st min for all four wavelengths during both events. However, Petrosian feature from IR and Red wavelengths only showed the highest level of statistical significance (*p* < 0.01) for both events (table 3 and figure 7*b*). Similarly, Petrosian feature from IR and Red also showed the highest level of statistical significance (*p* < 0.01) between 2nd min and recovery phases for CP stressor (table 3 and figure 7*b*). The differences in Petrosian feature were also found statistically significantly (*p* < 0.05) different between 2nd min and recovery phases for HG event only for Blue and IR wavelengths.

Average Higuchi value showed a consistent pattern (increase, decrease and increase during 1st min, 2nd min and recovery phase, respectively) for HG stressor for all wavelengths except Red. Similarly consistent but opposite pattern (decrease, increase, decrease) was found for CP stressor for the same set of wavelengths (Green, Blue and IR). Average Higuchi feature extracted from Red wavelength during CP and HG stressors showed (increase, increase and decrease) and (increase, decrease and decrease) patterns during 1st min, 2nd min and recovery phases, respectively. Changes in Higuchi feature values between base line and 1st min were found statistically significant for IR and Red wavelength and for both stressors (*p* < 0.05 for IR, *p* < 0.01 for Red). However, between 2nd min and recovery phases the change in Higuchi values were found statistically significant for IR during the CP event (*p* < 0.05) and Red during the HG event (*p* < 0.01) (table 3 and figure 7*c*).

### 3.3. Entropy features

**Figure 8.**
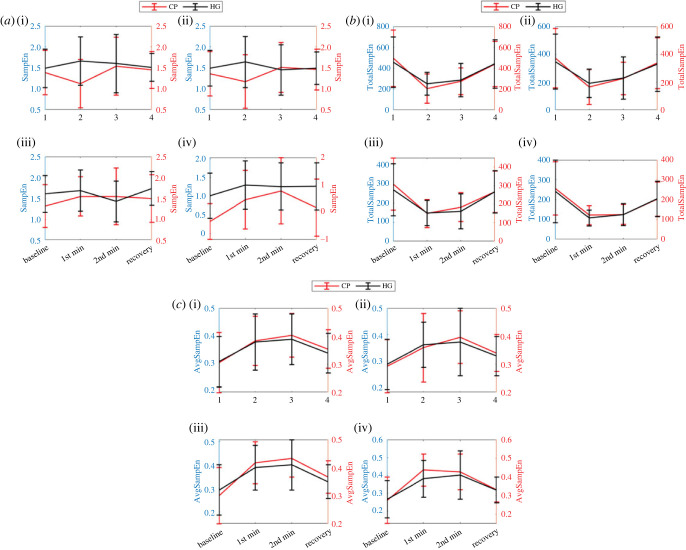
Pattern of (*a*) SampEn, (*b*) TotalSampEn and (*c*) AvgSampEn feature changes (during 1st min, 2nd min, recovery phase) for HG and CP stressor across the Green (i), Blue (ii), IR (iii) and Red (iv) wavelength.

Sample entropy (SampEn) presented in tables 1 and 2 has several empty entries, which means that there is at least one record for which SampEn is undefined. Thus, the significance results presented in table 3 were calculated using the defined values only. Similarly, the trend in variation of values presented in figure 8*a* was also plotted using those defined values of SampEn. Changes in average SampEn showed different patterns across phases for CP and HG stressors. Green and Blue wavelengths showed the same pattern (decrease, increase, decrease) for the CP event, whereas the opposite pattern (increase, decrease, increase) was found for Blue and IR wavelengths during the HG event. However, differences in SampEn values between differences phases of experiments were found statistically insignificant except for Green and Blue wavelengths during the HG event, where values between 1st min and 2nd min of stressor application were found statistically significantly different (*p* < 0.05).

Average TotalSampEn value showed a consistent pattern (decrease, increase and increase during 1st min, 2nd min and recovery phase, respectively) for both CP and HG stressors for all wavelengths. Differences in TotalSampEn values between baseline and 1st min as well as between 2nd min and recovery phases were found statistically highly significant (*p* < 0.01) during both events for all wavelengths except Blue (*p* < 0.05) during both events and Green (*p* < 0.05) during the HG event (table 3).

Similar to TotalSampEn, average AvgSampEn value showed a consistent but different pattern (increase, increase and decrease during 1st min, 2nd min and recovery phase, respectively) for both stressors and all wavelengths except the Red wavelength during CP, where the pattern found is increase, decrease and decrease. Differences in *AvgampEn* values between baseline and 1st min as well as between 2nd min and recovery phases were found statistically highly significant (*p* < 0.01) for most of the cases during both events (table 3). A few exceptions for which the statistical significance drops to *p* < 0.05 are: (i) between baseline and 1st min for Blue wavelength during CP event and (ii) between 2nd min and recovery phases for Green and Blue wavelengths during the HG event.

### 3.4. Area under the curve value

To further investigate which PPG wavelength is more sensitive with onset or withdrawal of the applied physical stressors, an area under the curve (AUC) value was calculated for onset (baseline versus 1st min during stressor application) and offset (2nd min versus recovery phase) of each stressor event (table 4). It is obvious that features extracted from IR and Red wavelengths showed higher (greater than or equal to 0.8) AUC for multiple features than other wavelengths to detect onset and offset of stressor events. These wavelengths showed AUC ≥ 0.8 value for detecting onset for both stressors for three features, namely Petrosian, TotalSampEn and AvgSampEn. Although the same set of features for Red wavelength showed a similar performance (AUC = 0.8) in offset detection for the CP event, only AvgSampEn showed the same performance for the IR wavelength. From the feature perspective, TotalSampEn showed more consistent performance across all PPG wavelengths during the CP event. On the other hand, both TotalSampEn and AvgSampEn showed consistent performance for IR and Red PPG wavelengths for both CP and HG events.

**Table 4.** Area under the ROC curve (AUC) of PPG features to detect difference in phases of a Cold Pressor and Hand Grip test. Let us define the phase differences as follows; *A* = Baseline versus 1st min and *C*—2nd min versus Recovery. AUC ranges between 0 and 1. The closer the value of AUC to 1, the higher the statistical significance of separation between the phases. AUC≥0.8 is highlighted in italics.

	CP				HG			
AC feature/event	Green	Blue	IR	Red	Green	Blue	IR	Red
phase difference	*A*, *C*	*A*, *C*	*A*, *C*	*A*, *C*	*A*, *C*	*A*, *C*	*A*, *C*	*A*, *C*
Mean	0.5, 0.5	0.6, 0.5	0.6, 0.5	0.6, 0.6	0.6, 0.5	0.6, 0.5	0.6, 0.6	0.5, 0.5
s.d.	0.6, 0.5	0.5, 0.5	0.6, 0.5	0.6, 0.6	0.6, 0.5	0.6, 0.5	0.6, 0.6	0.5, 0.5
Katz	0.7, 0.5	0.6, 0.5	0.6, 0.5	0.5, 0.6	0.5, 0.5	0.6, 0.6	0.6, 0.7	0.6, 0.5
Petrosian	0.7, 0.6	0.7, 0.6	*0.8*, 0.7	*0.8*, *0.8*	0.7, 0.6	0.7, 0.6	0.7, 0.7	*0.8*, 0.6
Higuchi	0.5, 0.5	0.5, 0.5	0.7, 0.7	0.7, 0.6	0.6, 0.5	0.5, 0.5	0.7, 0.6	0.7, 0.7
SampEn	0.6, 0.5	0.6, 0.6	0.6, 0.6	*0.8*, 0.6	0.6, 0.5	0.5, 0.5	0.6, 0.6	0.6, 0.5
TotalSampEn	*0.8*, 0.7	*0.8*, 0.7	*0.9*, 0.7	*0.9*, *0.8*	*0.8*, 0.7	0.7, 0.6	*0.8*, *0.8*	*0.9*, *0.8*
AvgSampEn	0.7, 0.7	0.7, 0.7	*0.8*, *0.8*	*0.9*, *0.8*	0.7, 0.6	0.7, 0.7	*0.8*, 0.7	*0.8*, 0.7

## 4. Discussion

PPG is a ubiquitous and cost-effective method of detecting blood volume changes in the skin, which, given that a reduction in blood volume indicates sympathetically mediated vasoconstriction, is an indirect indication of SA. This makes it an appealing candidate for non-invasive and continuous measure of SA in real-world environments on a continuous basis. However, before developing an SA measurement tool or device, several design issues need to be investigated thoroughly to understand their sensitivity to variations of SA. This includes the effect of a PPG wavelength and the choice of features.

In this study, we have investigated the sensitivity of statistical, fractal and nonlinear features extracted from the pulsatile component of the PPG from four different wavelengths (Green, Blue, IR and Red) during SA induced by physical stressor events—Cold Pressor and sustained Hand Grip. We have investigated the ability of different features in detecting onset (start of application of stressor) and offset (recovery phase) of the stressor events.

A synopsis of our findings is listed below:

— A larger set of features extracted from IR and Red PPG wavelengths were statistically significant in detecting physical stressor events as compared to Green and Blue wavelengths.
— TotalSampEn feature was found to be the most consistent in detecting stressor events across all PPG wavelengths.
— Three features based on Entropy and FD performed better in identifying onset of the applied physical stressor events for IR and Red wavelength.
— TotalSampEn extracted from all wavelengths showed high AUC (≥0.8) in detecting onset of a stressor event.
— Maximum AUC value obtained for onset detection is 0.9, in contrast to 0.8 for the offset.

In this study, the pulsatile (AC) component of the PPG signal was used for extracting features instead of the raw PPG signal. This is motivated by the fact that the AC component represents the cardiac synchronous variations in blood volume and is significantly influenced by sympathetic activation [8,31,32]. Due to the short segment length of analysis, we extracted a small set of statistical and nonlinear features that are suitable for short-length signals.

The Mean and s.d. features represented the magnitude and variation in AC amplitude, and none of these features showed sensitivity towards the application of stressors (table 4).

The features from the Fractal Dimension family measure the dynamics and complexity of AC amplitude time series. These features extract the hidden information contained in AC time series using fractals, which despite scaling, preserve the structure and shape of complex signals [33,34]. Out of three measures of complexity, the Petrosian feature showed statistically significant variation during onset of applied physical stressors (table 4 and figure 7*b*). However, only the Red wavelength showed 0.8 AUC area in detecting onset. This indicates that the application of physical stressors affects the dynamics of AC time series but not all wavelengths are equally sensitive in measuring those variations. It should be noted that the result of the FD analysis in this study might be affected by the smaller number of samples (approx. 120 samples/window). However, such an effect might not be severe [22].

The final set of features (SampEn, TotalSampEn, AvgSampEn) used in this study was extracted based on nonlinear KS-entropy family that measures irregularity of AC time-series signal. Although SampEn is efficient in dealing with short-term data, its dependence on input parameters affects the quality of information retrieval to a great extent [28]. The missing values in table 3 can be attributed to this unreliability of SamplEn measurement. This limitation was addressed by our group, where we proposed a data-driven approach for measuring entropy from short-length signals. We have extracted TotalSampEn and AvgSampEn features using this approach, which also estimates total and average irregularity in the AC time-series signal [26,28]. From the results, it is evident that these two features were most sensitive to stress-induced variations in AC time-series signals. This indicates that the irregularity in the time series of the PPG AC amplitude changes significantly with the application of physical stressors. In addition, figure 8*b* indicates that SA increases the regularity of the AC time-series signal by reducing the beat-to-beat variation in vascular tone. This is aligned with previously reported findings that stress leads to an increase in predictability or regularity and reduced complexity in heart rate variability signal [35]. Figure 8*b*,*c* is opposite due to the data-driven approach used by the entropy profiling technique, where the number of *r* values for which entropy values are calculated is determined from the signal itself. Therefore, if the signal is more regular, then it results in a reduced number of *r* values and vice versa. From the figure, we can assume that during the stress event, randomness decreases, which results in a reduced number of unique *r* values, which results in higher average entropy values during the stress event.

Since the AC time series also presents the modulation of heart rate variability, it is expected that the irregularity in the AC time series should reduce with the application of physical stressors [36].

Several previous studies reported the effect of the Cold Pressor test on blood pressure reactivity, pain tolerance, improving mental health and sympathetic nerve activity [12,37–39]. Karthik *et al.* used PTT for the assessment of SNA during CP events, which requires multi-site PPG acquisition. By contrast, we investigate a single site PPG measurement, which is better suited to monitoring SA in real-world environments on a continuous basis. In addition, the relative performance of multiple wavelengths helps to select the best wavelength and corresponding feature set that can be used for wearable device design for physical and psychological stressors. By contrast to existing literature, we have used two different stressors (CP and HG) for SA assessment, and the consistent performance across them further validates the reliability of studied features (tables 3 and 4; figure 8*b*).

## 5. Conclusion

Detectable changes in skin blood volume are an indirect indication of SA, leading to cutaneous vasoconstriction in humans. Photoplethysmography, an easy and cost-effective method to measure blood volume changes, is a promising candidate to facilitate quantification of sympathetic activity. In this study, we investigate the feasibility of using multi-wavelength PPG to extract information that correlates with SA. We have studied the impact of two physical stressors; the Cold Pressor test and Hand Grip exercise on 32 healthy individuals by measuring their respective non-invasive PPGs using Green, Blue, Infrared and Red wavelengths.

Statistical and nonlinear features were extracted from the pulsatile component of PPG. Our study has revealed that nonlinear especially entropy features from the PPG, specifically AverageSampleEn and TotalSampleEn, are the most promising candidates to retrieve correlating information pertaining to SA from physical stressors. Given the results of this study, we hypothesize that IR (Infrared) and Red wavelengths will perform best in detecting physical stressors. A similar study shall be carried out for mental/psychological stressors. In future, we aim to build a machine learning model using a combinationf of nonlinear features studied here to predict stress.

An accurate estimation of SA from PPG alone has the potential to enable objective quantification of a person’s response to a stressor. Coupled with understanding the person’s exposure and appraisal of stress, evidence-based stress management and psychological interventions can be developed at scale [1,4]. Future studies with larger cohorts and longer continuous recording are necessary to further validate the findings of this study and explore additional potentials of PPG signal for stress detection.

## Data Availability

Data and code are available on Zenodo at https://doi.org/10.5281/zenodo.7310705 [40].

## Footnotes

1 Verisense pulse+ the first line extension for its verisense^®^ wearable sensing platform—verisense. 2021.
